# PKC**ε** Phosphorylates and Mediates the Cell Membrane Localization of RhoA

**DOI:** 10.1155/2013/329063

**Published:** 2013-09-29

**Authors:** Tizhi Su, Samuel Straight, Liwei Bao, Xiujie Xie, Caryn L. Lehner, Greg S. Cavey, Theodoros N. Teknos, Quintin Pan

**Affiliations:** ^1^Arthur G. James Cancer Hospital and Richard J. Solove Research Institute, The Ohio State University Comprehensive Cancer Center, Columbus, OH 43210, USA; ^2^Department of Otolaryngology-Head and Neck Surgery, The Ohio State University Wexner Medical Center, Columbus, OH 43210, USA; ^3^Center for Live-Cell Imaging, Department of Microbiology and Immunology, University of Michigan, Ann Arbor, MI 48109, USA; ^4^Division of Hematology and Oncology, Department of Internal Medicine, University of Michigan Medical School, Ann Arbor, MI 48108, USA; ^5^Van Andel Research Institute, Grand Rapids, MI 49503, USA; ^6^Southwest Michigan Innovation Center, Kalamazoo, MI 49008, USA

## Abstract

Protein kinase C**ε** (PKC**ε**) signals through RhoA to modulate cell invasion and motility. In this study, the multifaceted interaction between PKC**ε** and RhoA was defined. Phosphopeptide mapping revealed that PKC**ε** phosphorylates RhoA at T127 and S188. Recombinant PKC**ε** bound to recombinant RhoA in the absence of ATP indicating that the association between PKC**ε** and RhoA does not require an active ATP-docked PKC**ε** conformation. Activation of PKC**ε** resulted in a dramatic coordinated translocation of PKC**ε** and RhoA from the cytoplasm to the cell membrane using time-lapse fluorescence microscopy. Stoichiometric FRET analysis revealed that the molecular interaction between PKC**ε** and RhoA is a biphasic event, an initial peak at the cytoplasm and a gradual prolonged increase at the cell membrane for the entire time-course (12.5 minutes). These results suggest that the PKC**ε**-RhoA complex is assembled in the cytoplasm and subsequently recruited to the cell membrane. Kinase inactive (K437R) PKC**ε** is able to recruit RhoA to the cell membrane indicating that the association between PKC**ε** and RhoA is proximal to the active catalytic site and perhaps independent of a PKC**ε**-RhoA phosphorylation event. This work demonstrates, for the first time, that PKC**ε** phosphorylates and modulates the cell membrane translocation of RhoA.

## 1. Introduction

Numerous publications have clearly defined the role of PKC*ε* as transforming oncogene in fibroblasts and epithelial cells. overexpression of PKC*ε* in NIH3T3 fibroblasts and FRC/TEX CL D rat colonic epithelial cells was shown to increase cell proliferation, enhance anchorage-independent colony formation, and induce a highly tumorigenic *in vivo* phenotype with tumor incidence of 100% [1, 2]. In addition, NIH3T3 fibroblasts with PKC*ε* overexpression were invasive and displayed a polarized morphology with extended long cellular membrane protrusions [3]. Epidermis-specific PKC*ε* transgenic mice developed highly malignant and metastatic squamous cell carcinomas in response to 12-O-tetradecanoylphorbol-13-acetate stimulation [4]. PKC*ε* was demonstrated to transform androgen-dependent LNCaP prostate cancer cells into an androgen-independent variant [5]. Moreover, transgenic mice with selective overexpression of PKC*ε* in the prostate epithelium developed prostate hyperplasia and prostate intraepithelial neoplasia [6]. Our laboratory demonstrated that inhibition of PKC*ε* in MDA-MB231 cells, a highly metastatic breast cancer cell line with elevated PKC*ε* levels, was sufficient to dramatically decrease *in vivo* tumor growth and reduce the incidence of lung metastasis [7]. Subsequently, PKC*ε* was shown to promote an invasive and motile phenotype in HNSCC through modulation of RhoA presumably through posttranslation phosphorylation [8].

RhoA, a member of the Rho GTPase family, has been implicated to be involved in the development and/or progression of numerous cancers. A recent report showed that overexpression of RhoA is sufficient to immortalize human mammary epithelial cells [9]. Elevated RhoA is associated with invasive breast cancer progression [10]. Moreover, miR-31 was reported to be inversely associated with metastasis through inhibition of RhoA in breast cancer patients [11]. Multivariate analysis revealed that elevated RhoA is an independent prognostic biomarker of poorer overall survival in pancreatic adenocarcinoma [12]. High levels of RhoA correlated with venous invasion, advanced pTNM stage, and prognosis in hepatocellular carcinoma [13, 14]. Increased RhoA is associated with tumor progression in ovarian carcinoma and lymph node metastasis and overall survival in bladder carcinoma [15, 16]. Similarly, RhoA was shown to be biomarker for lymph node metastasis and overall survival in esophageal squamous cell carcinoma [17]. RhoA, Rac2, and Cdc42 were found to be elevated in premalignant dysplastic and HNSCC cell lines in comparison to normal keratinocytes [18]. Furthermore, based on their immunohistochemistry analyses, RhoA was suggested to be a promising biomarker of malignancy and/or aggressiveness in head and neck squamous cell carcinoma (HNSCC) [18].

Our previous work provided the initial evidence linking two proteins, PKC*ε* and RhoA, intimately involved in metastasis. PKC*ε* was shown to signal through RhoA to modulate cell invasion and motility in HNSCC [8]. In this study, we further studied the interaction between PKC*ε* and RhoA. PKC*ε* was shown to phosphorylate RhoA at T127 and S188. Interestingly, an active ATP-docked PKC*ε* conformation is not required for PKC*ε* to bind to RhoA indicating that the PKC*ε*-RhoA complex is assembled independently of the transient substrate-kinase interaction at the catalytic site of PKC*ε*. Stoichiometric FRET analysis with HEK293 cells overexpressing mCherry-PKC*ε* and eGFP-RhoA revealed that the PKC*ε*-RhoA complex is assembled in the cytoplasm and subsequently translocates to the cell membrane. Our work revealed that PKC*ε* phosphorylates RhoA but, intriguingly, also has a kinase-independent action to function as a chaperone to traffic RhoA to the cell membrane.

## 2. Materials and Methods

### 2.1. Plasmid Constructs

Human PKC*ε* cDNA was cloned into pENTR/D-TOPO vector (Invitrogen, Carlsbad, CA) by PCR from a human cDNA library (Clontech, Mountain View, CA). The N-mCherry-tagged PKC*ε* was made by inserting PKC*ε* open reading frame into BglII/XbaI site of mCherry-C1 vector (Clontech, Mountain View, CA). mCherry-PKC*ε*/K437R mutant was generated using the QuikChange Lightning kit (Agilent Technologies, Inc., Santa Clara, CA). The positive control plasmid mCherry-linker-eGFP for stoichiometric FRET analysis was made by inserting mCherry DNA fragment into NheI/BglII sites and followed by eliminating the sequence between BamH1 and BglII sites within the multiple cloning site in vector eGFP-C1 (Clontech), resulting in a 10 amino acid long in-frame linker SGLKDPPVAT.

### 2.2. Cell Line

HEK293 cells were purchased from ATCC (Rockville, MD) and cultured in Dulbecco's modified Eagle's medium supplemented with penicillin (100 units/mL), streptomycin (100 *μ*g/mL), and 10% fetal bovine serum. 

### 2.3. *In Vitro* Kinase Assay

Recombinant PKC*ε* was incubated with recombinant RhoA in kinase buffer (24 mM Tris (pH 7.4), 0.5 mM EDTA, 0.5 mM EGTA, 10 mM *β*-mercaptoethanol, 1 *μ*g/mL leupeptin, 1 *μ*g/mL aprotinin, and 50 *μ*g/mL PMSF) containing PKC activators, phosphatidylserine and diacylglycerol, and [^32^P]ATP for 30 minutes at 25°C. Subsequently, termination buffer consisting of 7.5 M guanidine-HCl was added to stop the reaction. The incubation reaction was separated by SDS-PAGE and visualized using autoradiography.

### 2.4. Phosphopeptide Mapping

RhoA was phosphorylated by PKC*ε* *in vitro* and then subjected to digestion by trypsin, chymotrypsin, or Glu-C. Following enzyme digestion the sample was acidified to 0.5% trifluoroacetic acid concentration and stored at −20°C until further analyzed. The digested RhoA protein was analyzed by reverse-phase nanoscale LC-MS^E^ using a Waters QTof Premier mass spectrometry system. Prior to analysis EDTA and diammonium phosphate were added to sample for a final concentration of 25 mM each, and 11–25 ng of digested protein was analyzed. Peptides were separated using acetonitrile/water mobile phases containing 0.1% formic acid on a Waters NanoAcquity UPLC system employing a 300 *μ*m ID × 20 mm C-18 5 *μ*m particle Symmetry trap column and a 75 *μ*m ID × 150 mm C-18 1.7 *μ*m BEH analytical column. Peptides were trapped for 15 minutes at 3 *μ*L/min followed by gradient elution using 0–28% acetonitrile in 40 minutes through the analytical column at 300 nL/min. ESI was conducted at approximately 3.3 kV using in-house prepared spray emitters. Emitters were constructed by sleeving a 7 cm piece of 20 *μ*m ID × 90 OD FSC into a 3 cm piece of 100 *μ*m ID × 360 OD FSC and gluing the junction with epoxy. The polyimide coating on the terminal end of the emitter was burned off using a microtorch, and the emitter was used with a Waters NanoEase ESI mount. The Qtof Premier mass spectrometer was programmed to collect alternate scan MS^E^ data as previously described [19, 20]. Briefly, MS^E^ data collection was performed by a low collision energy acquisition of 0.8 seconds followed by a high collision energy acquisition for 0.8 seconds without quadrupole mass filtering across a 50–1990 m/z mass range. This was performed in an alternating fashion during a 65-minute run and termed LC-MS^E^ analysis. Low collision energy acquisition records all peptide precursor mass data, while the high collision energy portion of the acquisition collected peptide fragmentation data. Following the low collision energy acquisition set at 10 volts, collision energy was ramped from 10 volts to 40 volts over the 0.8 second high collision energy acquisition to accommodate peptides requiring different collision energy for fragmentation. Glu-fibrinopeptide at a concentration of 200 fmol/*μ*L in 25% acetonitrile/water/0.1% formic acid was introduced as a lockspray calibrant through a second ESI probe at 0.5 *μ*L/min using an auxiliary UPLC pump. Lockspray data was collected for 1 second every 30 seconds over a 65-minute analysis. 

Following an LC-MS^E^ analysis, data processing was performed using Waters PLGS software version 2.3 build 23 using the following parameters: low-energy threshold 100 counts, high-energy threshold 10 counts, and an intensity threshold of 1000 counts. Data processing combined the signal intensity of all charge states generated from a given peptides into singly charged MH^+^ values and determined the peak apex for both low-energy precursors and all fragment ions within the vicinity of the precursor. This lockmass corrected, accurate mass data was used in two ways. First, the data was used to search Human RefSeq database version 17 within PLGS software using the following search parameters: peptide and fragment tolerance was automatic, the minimum fragment Ion matches per peptide were 3, the minimum fragment ions per protein were 7, the minimum peptides matches per protein were 1, missed cleavages were 2, the false positive rate was 4%, and modifications allowed were Acetyl N-term, Carbamidomethyl-C, Carbamyl N-term, and phosphorylation at STY. Second, low-energy precursor MH^+^ data was copied into Excel and compared to MH^+^ values calculated for predicted RhoA trypsin, chymotrypsin, or Glu-C protease peptides bearing up to 4 phosphate groups. (Lighthouse Data, GPMAW version 8.00sr1, Odense, Denmark). Experimental MH^+^ masses that matched within 0.03 Da of GPMAW calculated values were evaluated manually in PLGS or Masslynx Protein/Peptide Editor software. Possible phosphopeptide assignment was made when the measured mass was within 0.03 Da of the calculated phosphopeptide mass and greater than 3 accurate mass product ions could be assigned to a peptide sequence. Confident site-specific phosphorylation also used this criteria but further required fragment ions including phosphorylated serine or threonine amino acids.

### 2.5. Immunoprecipitation

Recombinant PKC*ε* (GenWay Inc., San Diego, CA) was incubated with recombinant RhoA (Cytoskeleton, Denver, CO) in PKC kinase buffer for 30 minutes at 25°C. The binding reaction was immunoprecipitated using agarose-conjugated anti-PKC*ε* antibody or IgG (Abcam, Cambridge, MA) at 4°C with gentle agitation overnight. The suspension was centrifuged at 1,000 ×g for 1 minute, and the agarose beads were washed three times with ice-cold PBS and resuspended in SDS sample buffer. The same procedure was performed to immunoprecipitate the binding reaction containing His-tagged PKC*ε*-kinase domain (BioBasic Inc., Markham, Canada), and RhoA except agarose-conjugated anti-His antibody (Abcam) was used. The immunoprecipitated samples were boiled in SDS sample buffer, resolved by SDS-PAGE, and transferred to Immobilon membrane. The membranes were incubated with anti-RhoA (Cytoskeleton) or anti-His (Millipore, Billerica, MA) antibodies and visualized by ECL using the Fast Western kit (Pierce, Rockford, IL).

### 2.6. Fluorescence Microscopy and Quantitative Stoichiometric FRET Analysis

HEK293 cells were seeded on 35 mm glass-bottomed dishes one day prior to transfection with mCherry-PKC*ε* and eGFP-RhoA. Fluorescence microscopy experiments were performed in the Center for Live-Cell Imaging (CLCI) at the University of Michigan Medical School using an Olympus IX70 inverted microscope (Olympus, Center Valley, PA). Experiments involving live-cell imaging employed a heated stage (Harvard Apparatus, Inc., Holliston, MA) in combination with HEPES-buffered medium to maintain cell viability and activity for several hours of microscopic observation. Illumination was provided from a 100 W halogen lamp for phase-contrast microscopy and by an X-Cite 120 metal halide light source (EXFO, Mississauga, Canada) for fluorescent microscopy. The microscope was equipped with 100x (oil immersion; UPlan Fl, NA = 1.30), 40x (LCPLanFl, NA = 0.6), and 10x (CPlan, NA = 0.25) objectives. Excitation and emission filter sets (Chroma Technology Corp., Rockingham, VT) were used for fluorescent imaging; in particular set number 86014v2 includes filters used for GFP (excitation 492 nm/BP18, emission 535 nm/BP40) and mCherry (excitation 572 nm/BP23, emission 630 nm/BP60). The excitation and emission filters were mounted in a Lambda 10-3 automatic filter wheels (Sutter Instrument Co., Novato, CA) allowing rapid filter switching. Images were collected using a CoolSNAP HQ2 14-bit CCD camera (Photometrics, Tucson, AZ). All devices were controlled through Metamorph Premier v7.7 software (Molecular Devices, Downingtown, PA). Quantitative analysis of the imaging data and the preparation of presentation quality images were performed using Metamorph v7.7 software. Quantitative stoichiometric FRET analysis of the data was performed with proprietary software created by the CLCI staff using MATLAB R2009a (The Mathworks, Natick, MA), and this FRETcalc software can be obtained from the University of Michigan Tech Transfer. The methods and algorithms used in FRET stoichiometry have been previously described [21, 22]. 

## 3. Results and Discussion

### 3.1. Results

#### 3.1.1. PKC*ε* Phosphorylates and Binds to RhoA

**Figure 1:**
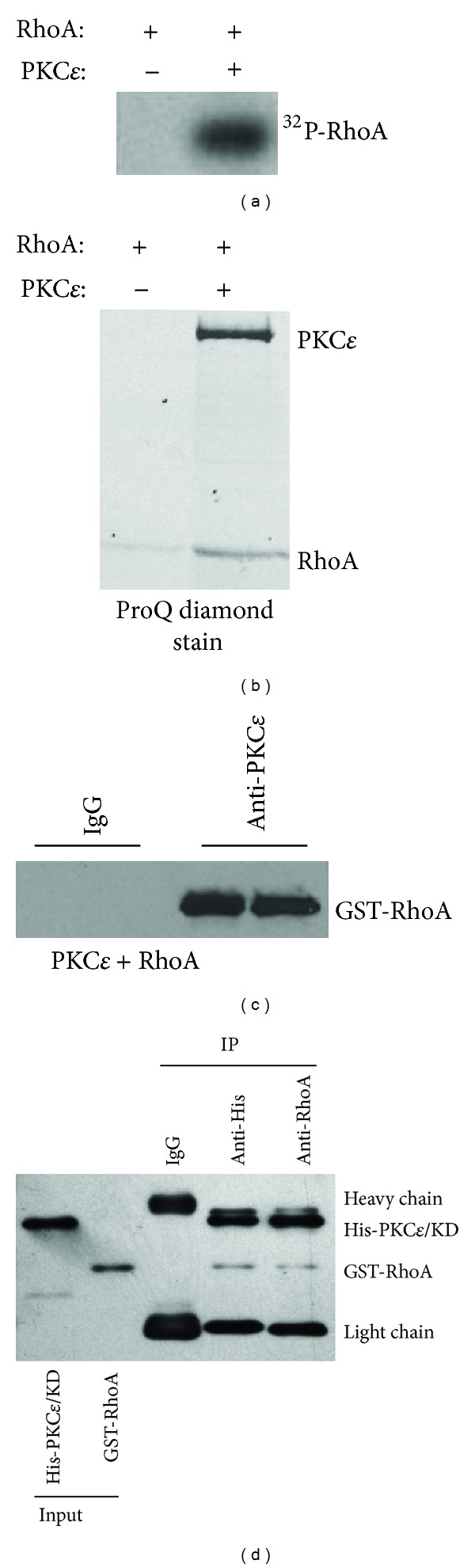
PKC*ε* phosphorylates and binds to RhoA. (a) PKC*ε* phosphorylates RhoA. Recombinant RhoA was incubated with or without recombinant PKC*ε* in kinase buffer containing PKC activators, phosphatidylserine and diacylglycerol, and [^32^P]ATP for 30 minutes at 25°C. Subsequently, the incubation reaction was terminated, separated by SDS-PAGE and visualized using autoradiography. (b) Pro-Q Diamond staining of phosphorylated RhoA. Recombinant RhoA was incubated with or without recombinant PKC*ε* in kinase buffer containing PKC activators, phosphatidylserine and diacylglycerol, and ATP for 30 minutes at 25°C. Subsequently, the incubation reaction was terminated, separated by SDS-PAGE and visualized using a stain specific to phosphoproteins. (c) PKC*ε* binds to RhoA. Recombinant PKC*ε* was incubated with recombinant RhoA in kinase buffer containing PKC activators, phosphatidylserine and diacylglycerol, for 30 minutes at 25°C. The binding reaction was immunoprecipitated using agarose-conjugated anti-PKC*ε* or nonspecific IgG antibody. The immunoprecipitated proteins were visualized by western blot analysis using an anti-RhoA antibody. Two independent immunoprecipitation experiments are presented. (d) The kinase domain of PKC*ε* binds to RhoA. Recombinant His-tagged PKC*ε*-kinase domain was incubated with recombinant GST tagged-RhoA in kinase buffer containing PKC activators, phosphatidylserine and diacylglycerol, for 30 minutes at 25°C. The binding reaction was immunoprecipitated using agarose-conjugated anti-His, anti-RhoA, or nonspecific IgG antibody. The immunoprecipitated proteins were visualized by western blot analysis using an anti-His and anti-RhoA antibody.

Our laboratory reported that PKC*ε* modulates RhoA activity in HNSCC presumably through posttranslation phosphorylation [8]. *In silico* prediction of phosphorylation sites identified multiple serine and threonine residues that are putative PKC phosphorylation sites on RhoA suggesting that direct phosphorylation of RhoA through PKC*ε* may be a possibility. To determine if PKC*ε* can directly phosphorylate RhoA, we performed an* in vitro* kinase reaction and incubated recombinant PKC*ε* with RhoA in the presence of PKC activators, phosphatidylserine and diacylglycerol, and ^32^P-ATP. As shown in Figure 1(a), PKC*ε* directly phosphorylated RhoA. Pro-Q Diamond, a phosphoprotein staining reagent, confirmed RhoA as a substrate for PKC*ε* (Figure 1(b)). Next, we identified the phosphorylation sites on RhoA using phosphopeptide mapping with liquid chromatography-mass spectrometry/mass spectrometry (LC-MS/MS). Phosphorylated RhoA was digested with trypsin, chymotrypsin, or Glu-C. LC-MS^E^ analysis of peptides resulting from trypsin digested RhoA showed about 83% coverage, whereas the combined data from trypsin and Glu-C digested RhoA showed 100% coverage of the serine and threonine residues on RhoA. Thus, it is reasonable to conclude that a comprehensive phosphopeptide map of RhoA was generated using trypsin and Glu-C. Phosphopeptide analysis revealed T127 and S188 as the confident PKC*ε*-mediated phosphorylation sites on RhoA. Our results provide the first evidence that RhoA is a direct substrate for PKC*ε* phosphorylation.

#### 3.1.2. PKC*ε* Associates with RhoA

There is limited, although intriguing, literature demonstrating that a kinase preassembles with its substrate prior to a kinase phosphorylation event. The preassembled kinase-substrate complex not only increases specificity but also shortens the time between kinase activation and phosphorylation of the substrate [23, 24]. To determine if PKC*ε* preassembles with RhoA without an active ATP-docked PKC*ε* conformation, recombinant PKC*ε* and RhoA were incubated in ATP-free *in vitro* kinase buffer. As shown in Figure 1(c), PKC*ε* was able to bind to RhoA under this condition. Moreover, the kinase domain of PKC*ε* was sufficient to bind to RhoA demonstrating that the RhoA docking site is within the PKC*ε* kinase domain (Figure 1(d)). These results indicate that the binding between PKC*ε* and RhoA does not require an active ATP-docked PKC*ε* kinase conformation, and thus, the interaction between these two proteins is more complex than a transient substrate-kinase intermediate state.

#### 3.1.3. PKC*ε* Colocalizes with RhoA at the Cell Membrane in Response to PMA

**Figure 2:**
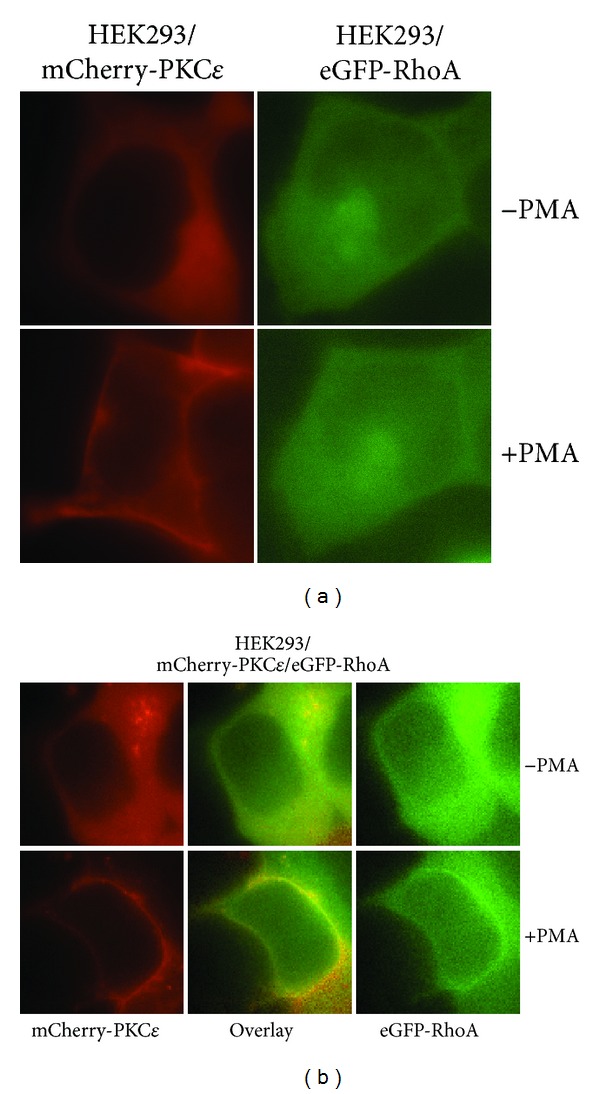
PKC*ε* colocalizes with RhoA at the cell membrane. (a) PKC*ε* translocates to the cell membrane, and RhoA remains localized at the cytoplasm in response to PMA. HEK293 cells were transfected with mCherry-PKC*ε* (left panel) or eGFP-RhoA (right panel). Fluorescence images were captured prior to and 15 minutes after PMA (100 nM) stimulation. (b) PKC*ε* colocalizes with RhoA at the cell membrane. HEK293 cells were cotransfected with mCherry-PKC*ε* and eGFP-RhoA. Fluorescence images were captured prior to and 15 minutes after PMA (100 nM) stimulation.

There is evidence that a preassembled kinase-substrate complex not only enhances phosphorylation specificity and efficiency but also plays a role in cellular localization [25]. To determine if PKC*ε* mediates the localization of RhoA, HEK293 cells were transfected with mCherry-PKC*ε* or eGFP-RhoA or cotransfected with mCherry-PKC*ε* and eGFP-RhoA. Subcellular localization of PKC*ε* and RhoA was visualized in living cells using fluorescence microscopy in the presence or absence of phorbol 12-myristate 13-acetate (PMA). Activation of PKCs with PMA is associated with the translocation of PKCs to the cell membrane. As expected, PKC*ε* was translocated from the cytoplasm to the cell membrane following PMA stimulation in HEK293 cells overexpressing mCherry-PKC*ε* (Figure 2(a)). In contrast, in HEK293 cells overexpressing eGFP-RhoA, RhoA remained at the cytoplasm following PMA treatment. As shown in Figure 2(b), PMA treatment induced RhoA to colocalize with PKC*ε* at the cell membrane in HEK293 cells transfected to overexpress mCherry-PKC*ε* and eGFP-RhoA. Our data showed that PKC*ε* traffics RhoA to the cell membrane following a general PKC activation signal in HEK293 cells.

#### 3.1.4. Dynamic Interaction between PKC*ε* and RhoA in Live Cells

**Figure 3:**
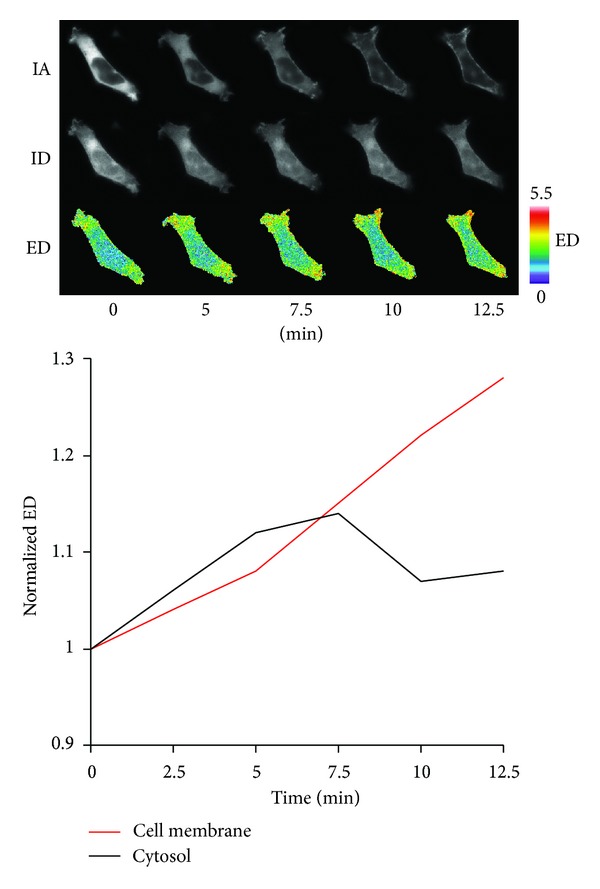
Stoichiometric FRET analysis of PKC*ε*-RhoA interaction in live cells. HEK293 cells were cotransfected with mCherry-PKC*ε* and eGFP-RhoA. Fluorescence images were captured prior to and every 15 seconds after PMA (100 nM) stimulation for 12.5 minutes and then subjected to quantitative FRET analysis. The images presented at each time point represent mCherry-PKC*ε* (acceptor image, IA), eGFP-RhoA (donor image, ID), and the FRET interaction between mCherry-PKC*ε* and eGFP-RhoA (ED). The color bars at the end of the panels indicate the scaling of the ED images with warmer colors representing higher values. Time course of normalized ED for the cell membrane and cytoplasm is presented. ED was normalized to the ED of the cytoplasm at the 0 minute time point. Cell membrane is defined as ±1 *μ*m from the cell membrane border. Cytoplasm is defined as all intracellular space, including the nucleus, 1 *μ*m from the cell membrane border.

To better define the interaction between PKC*ε* and RhoA with spatiotemporal resolution, HEK293 cells overexpressing mCherry-PKC*ε* and eGFP-RhoA were stimulated with PMA, and images collected over a 12.5 minute time course were subjected to quantitative stoichiometric FRET analysis. PMA treatment induced an obvious reorganization of mCherry-PKC*ε* and eGFP-RhoA in the cell from the cytoplasm to the cell membrane as evidenced by comparing the IA and ID images at 0 min and 12.5 min after PMA stimulation, respectively (Figure 3). Furthermore, PMA-treatment resulted in an overall increase in ED, a measure of the FRET efficiency of the interaction between mCherry-PKC*ε* and eGFP-RhoA. The initial increase and peak in ED occurred in the cytoplasm followed by an elevation of the PKC*ε*-RhoA interaction at the cell membrane for the entire time course. Recruitment of RhoA and the increase in FRET activity was especially robust in the actively ruffling regions of the cell (upper and bottom right corners of the cell). Taken together, FRET analysis demonstrated that in response to PMA stimulation, the PKC*ε*-RhoA complex is recruited to the cell membrane over time, and furthermore, the PKC*ε*-RhoA complex may be preassembled in the cytoplasm prior to translocation to the cell membrane.

#### 3.1.5. Kinase-Inactive PKC*ε* Colocalizes with RhoA at the Cell Membrane

**Figure 4:**
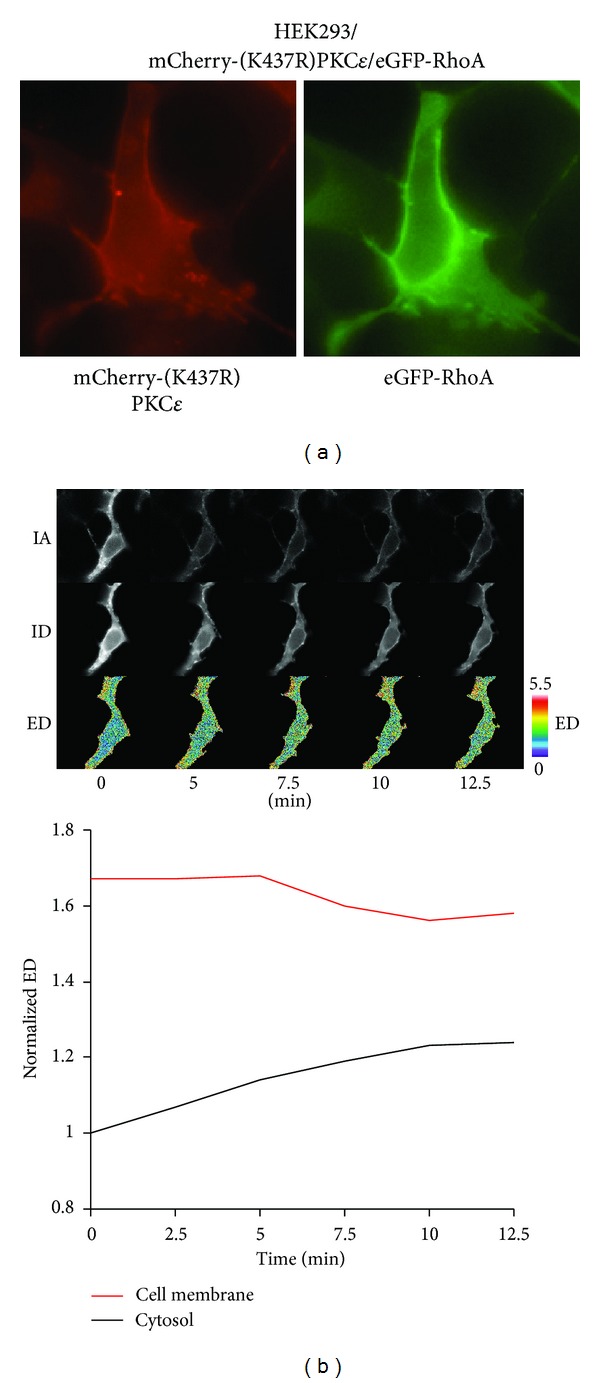
Kinase-inactive PKC*ε* colocalizes with RhoA at the cell membrane. (a) Colocalization of kinase-inactive PKC*ε* and RhoA. HEK293 cells were cotransfected with mCherry-PKC*ε*/K437R and eGFP-RhoA. Fluorescence images were captured prior to and 15 minutes after PMA (100 nM) stimulation. (b) Stoichiometric FRET analysis of kinase-inactive PKC*ε* and RhoA interaction. HEK293 cells were cotransfected with mCherry-PKC*ε*/K437R and eGFP-RhoA. Fluorescence images were captured prior to and every 15 seconds after PMA (100 nM) stimulation for 12.5 minutes and then subjected to quantitative FRET analysis. The images presented at each time point represent mCherry-PKC*ε*/K437R (acceptor image, IA), eGFP-RhoA (donor image, ID), and the FRET interaction between mCherry-PKC*ε*/K437R and eGFP-RhoA (ED). The color bars at the end of the panels indicate the scaling of the ED images with warmer colors representing higher values. Time course of normalized ED for the cell membrane and cytoplasm is presented. ED was normalized to the ED of the cytoplasm at the 0 minute time point. Cell membrane is defined as ±1 *μ*m from the cell membrane border. Cytoplasm is defined as all intracellular space, including the nucleus, 1 *μ*m from the cell membrane border.

Our *in vitro* protein binding experiments indicate that an active ATP-docked PKC*ε* confirmation is not required for PKC*ε* to bind to RhoA. The kinase-inactive PKC*ε* mutant (K437R) contains a point mutation in the ATP binding pocket to prevent ATP occupancy. Interestingly, PKC*ε*/K437R is localized to the cell membrane in unstimulated HEK293 cells overexpressing mCherry-PKC*ε*/K437R (Figure 4(a)). HEK293 cells cotransfected with mCherry-PKC*ε*/K437R and eGFP-RhoA showed colocalization of these two proteins at the cell membrane without PMA stimulation. In support of these observations, quantitative stoichiometric FRET analysis confirmed the interaction between mCherry-PKC*ε*/K437R and eGFP-RhoA at the cell membrane prior to PMA stimulation (Figure 4(b)). The FRET signal at the cell membrane did not change significantly after PMA treatment indicating that the PKC*ε*/K437R-RhoA molecules at that cell compartment were already in complex with each other. These results indicate that the PKC*ε*-RhoA interaction occurs in the absence of ATP and thus do not require an active catalytic site on PKC*ε*. The K437R mutation appears to alter the conformation of PKC*ε* to expose the critical amino acids required to interact with RhoA. Our data confirm that cell membrane localization of RhoA is mediated independently of a PKC*ε*-RhoA phosphorylation event.

### 3.2. Discussion

Regulation of RhoA activity is tightly controlled in the GDP/GTP cycle through the coordinated interactions between GTPase activating proteins (RhoGAPs), guanine dissociation inhibitors (RhoGDIs), and guanine nucleotide exchange factors (RhoGEFs). RhoGEFs activate RhoA by catalyzing the exchange of GDP to GTP. RhoGAPs deactivate RhoA by enhancing the intrinsic GTPase activity of RhoA to hydrolyze GTP to GDP. RhoGDIs prevent RhoA activation by sequestering GDP-bound RhoA in the cytoplasm. In addition to this well-described regulatory pathway, there is evidence that posttranslational phosphorylation is an alternate mechanism used to control RhoA. Protein kinase A (PKA) was reported to phosphorylate S188 of RhoA resulting in relocalization of GTP-bound RhoA from the membrane to the cytoplasm, possibly through enhanced interaction with RhoGDI [26]. Protein kinase G (PKG) activation was demonstrated to increase RhoA stability resulting in an increase in total RhoA protein levels [27]. Furthermore, phosphorylation of S188 on RhoA protected RhoA, particularly the GTP-bound active form, from ubiquitin/proteasome-mediated degradation [28]. In the present study, PKC*ε* was also shown to phosphorylate S188 of RhoA. It is interesting that three different kinases, PKA, PKG, and PKC*ε*, are able to phosphorylate S188 suggesting that phosphorylation of S188 may be a nondiscriminatory mechanism to enhance RhoA stability. In addition to S188, T127 was identified as a novel RhoA phosphorylation site suggesting that PKC*ε* may sequentially phosphorylate RhoA to fine tune RhoA levels and/or activation. Ongoing efforts in our laboratory will delineate the physiological significance of T127 and S188 phosphorylation on RhoA function in HNSCC. 

We made the novel observation that recombinant PKC*ε* associates with recombinant RhoA in the absence of ATP indicating that the PKC*ε*-RhoA complex is assembled without an active ATP-docked PKC*ε* conformation. Additionally, kinase-inactive PKC*ε* was sufficient to colocalize with RhoA at the cell membrane in live HEK293 cells providing further evidence that the PKC*ε*-RhoA interaction is much more involved than as a transient kinase-substrate transition state. These observations is consistent with a published report demonstrating that the mitogen activated protein kinase (MAPK) substrate complexes are often spatially separate from the kinase active site and the substrate phosphorylation site [23]. The region of the kinase that binds to a substrate has only been identified in two cases, for c-Jun aminoterminal kinase 2 (JNK2) and C-terminal Src kinase (Csk) [29, 30]. The substrate-docking sites for JNK2 and Csk were identified to be within the kinase domain and in proximity, within 50 amino acids, to the catalytic loop of the kinase [29, 30]. Consistent with these results, the kinase domain of PKC*ε* is sufficient to bind to RhoA. Our work showed that PKC*ε* binds to RhoA within the kinase domain and without the requirement of ATP.

The accepted model of PMA-mediated activation of PKCs is that PMA changes PKCs from a closed to an open conformation resulting in translocation of PKCs to the cell membrane. FRET results showed that the initial response to PMA is an increase in the molecular interaction between PKC*ε* and RhoA in the cytoplasm. This observation indicates that the active PKC*ε* conformation is required to expose the RhoA docking site and thus allow RhoA to complex with PKC*ε*. The interaction between PKC*ε* and RhoA showed an early peak at the cytoplasm and then decreased to basal levels for the remainder of the time course. In contrast, a gradual but prolonged increase in FRET intensity was observed at the cell membrane, in particular the actively ruffling regions of the cell, over the entire time course. A plausible explanation is that, following a PKC activation signal, PKC*ε* and RhoA are assembled in the cytoplasm, and the resulting complex is subsequently trafficked to the cell membrane. It is important to point out that PMA does not induce translocation of RhoA to the cell membrane without the presence of PKC*ε*. Therefore, the recruitment of the PKC*ε*-RhoA complex to the cell membrane is completely dependent on the cellular localization of PKC*ε* in response to a stimulus. 

Fluorescence microscopy showed that kinase-inactive PKC*ε* is predominantly localized to the cell membrane under basal conditions. This result suggests that the PKC*ε*/K437R is in an open conformation capable to interact with chaperone proteins involved in PKC*ε* translocation. The cellular localization of RhoA is concentrated at the cell membrane in cells cotransfected with mCherry-PKC*ε*/K437R and eGFP-RhoA. Similarly, FRET analysis showed that the interaction between PKC*ε*/K437R and RhoA is concentrated at the cell membrane rather than at the cytoplasm in unstimulated cells. Furthermore, PMA did not alter the extent of the molecular interaction between PKC*ε*/K437R and RhoA at the cell membrane. FRET analysis with PKC*ε*/K437R confirmed our *in vitro* observations and showed that PKC*ε* is able to recruit RhoA to the cell membrane without a PKC*ε*-RhoA phosphorylation event. Taken together, these findings support a kinase independent role of PKC*ε* as a chaperone to traffic RhoA to the cell membrane. 

## 4. Conclusions

Work from our laboratory provided the initial evidence linking two proteins, PKC*ε* and RhoA, intimately involved in metastasis. The PKC*ε*-RhoA signaling module was shown to modulate cancer cell invasion and motility. However, the molecular mechanism of PKC*ε* regulation of RhoA remains to be elucidated. In this study, our results revealed that PKC*ε* has both kinase-dependent and kinase-independent functions to regulate RhoA; PKC*ε* directly phosphorylates RhoA and, moreover, serves as a chaperone to translocate RhoA to the cell membrane.
